# Iatrogenic esophageal dysmotility as a barrier to transplantation in pulmonary arterial hypertension

**DOI:** 10.1016/j.jhlto.2024.100098

**Published:** 2024-04-20

**Authors:** Michael S. Miller, Shelsey W. Johnson, Alexander R. Opotowsky, Michael J. Landzberg, Nirmal S. Sharma, Hilary J. Goldberg, Alexandra K. Wong, Alison S. Witkin, Josanna Rodriguez-Lopez, Ronald H. Goldstein, Bradley A. Maron, Bradley M. Wertheim

**Affiliations:** a Department of Medicine, Brigham and Women’s Hospital and Harvard Medical School, Boston, Massachusetts; b Division of Pulmonary and Critical Care Medicine, Department of Medicine, Brigham and Women’s Hospital and Harvard Medical School, Boston, Massachusetts; c Department of Pulmonary, Allergy, Sleep, and Critical Care Medicine, Veterans Affairs Boston Healthcare System, Boston, Massachusetts; d Department of Pediatrics, University of Cincinnati School of Medicine, Cincinnati, Ohio; e Adult Congenital Heart Program, Heart Institute, Cincinnati Children’s Hospital Medical Center, Cincinnati, Ohio; f Division of Cardiovascular Medicine, Department of Medicine, Brigham and Women’s Hospital and Harvard Medical School, Boston, Massachusetts; g Department of Pediatrics, Boston Children’s Hospital, Boston, Massachusetts; h Department of Medicine, Division of Pulmonary and Critical Care Medicine, Massachusetts General Hospital and Harvard Medical School, Boston, Massachusetts; i The Pulmonary Center, Division of Pulmonary, Allergy, Sleep and Critical Care Boston University School of Medicine, Boston, Massachusetts; j Department of Medicine, University of Maryland School of Medicine, Baltimore, MD and the University of Maryland-Institute for Health Computing, Bethesda, Maryland

**Keywords:** pulmonary arterial hypertension, phosphodiesterase-type 5 inhibitor, esophageal dysmotility, lung transplantation

## Abstract

Esophageal dysmotility is identified as a contraindication to lung transplantation at some centers due to increased risks of acute rejection, pulmonary infection, and chronic lung allograft dysfunction. Phosphodiesterase-type 5 inhibitors (PDE5i) are a cornerstone pharmacotherapy for pulmonary arterial hypertension (PAH) and are known to exert off-target effects that may impact lung transplant candidacy, including impaired esophageal contractility and decreased lower esophageal sphincter tone. We report 2 patients with PAH who were initially declined listing for lung transplantation due to iatrogenic esophageal dysmotility induced by PDE5is. Upon discontinuation of PDE5i therapy, these patients experienced significant improvement in esophageal motility within 14 days and met the criteria for transplant listing at their centers. Recognizing and mitigating the off-target effects of PDE5i medications is critical for maximizing access to transplant for patients with PAH.

Lung transplantation is indicated for refractory pulmonary arterial hypertension (PAH). Patients with PAH experience higher rates of transplant waitlist mortality, lower likelihood of transplantation, and higher post-transplant mortality compared to other lung diseases.^1^, ^2^ Therefore, identifying PAH-specific barriers to transplant has important implications for management in end-stage PAH.^1^ Esophageal dysmotility following lung transplantation increases the risk of acute rejection, pulmonary infection, and chronic lung allograft dysfunction, and is identified as a contraindication to transplantation at some centers that include high-resolution esophageal manometry as part of pretransplant risk assessment.^3^

Phosphodiesterase-type 5 inhibitors increase bioavailable levels of nitric oxide and are a cornerstone pharmacotherapy for PAH. However, PDE5i medications are also associated with off-target effects that may be important for lung transplant candidacy, including impaired esophageal contractility and decreased lower esophageal sphincter (LES) tone.^4^, ^5^, ^6^ We hypothesized that PDE5i use is associated with esophageal dysmotility in patients with PAH and may have implications for lung transplant candidacy. This study was approved by the institutional review board at the Mass General Brigham Health System (2022P001026). Using the Mass General Brigham Research Patient Data Registry platform, we retrospectively identified patients at Brigham and Women’s Hospital (BWH) and Massachusetts General Hospital in Boston, Massachusetts with catheterization-proven PAH on PDE5i therapy, in the absence of connective tissue diseases, who underwent clinically indicated esophageal manometry. Of 30 patients meeting these criteria, 17 (56.7%) were found to have evidence of impaired esophageal motility on manometry (N = 12 sildenafil, N = 5 tadalafil). Analysis of transplant evaluations at Brigham and Women’s Hospital between 2015 and 2023 identified 14 patients with PAH and without connective tissue disease who were declined for lung transplantation specifically due to manometry results (Table 1). Of these patients, 9 (64.3%) were on PDE5i. Two patients underwent repeat manometry following discontinuation of PDE5i, which led to favorable changes in esophageal function and transplant candidacy.

**Table 1:** Demographics of Patients With PAH Who Were Declined for Lung Transplantation Based on Esophageal Dysmotility

Baseline characteristics	*n* = 14
Age – mean (SD)	53.4 (14.2)
Female – no (%)	4 (29.0)
Hemodynamic profiles	
Right atrial pressure, mm Hg – mean (SD)	8.1 (4.5)
Mean pulmonary artery pressure, mm Hg – mean (SD)	40.0 (12.7)
Pulmonary artery wedge pressure, mm Hg – mean (SD)	10.9 (3.0)
Cardiac index liter/min/m^2^ – mean (SD)	2.5 (0.5)
Pulmonary vascular resistance dynes/sec/cm^5^ – mean (SD)	558 (322)
Pulmonary hypertension medications	
Phosphodiesterase 5 inhibitors – no (%)	9 (64.3)
Sildenafil – no (%)	6 (42.9)
Tadalafil – no (%)	3 (21.4)
Riociguat – no (%)	1 (7.1)
Endothelin receptor antagonists – no (%)	5 (35.7)
Prostacyclin analogs – no (%)	8 (57.1)

Abbreviations: No, number; SD, standard deviation.

Nine out of 14 patients (64.3%) were found to be on PDE5i at the time of esophageal manometry.

## Case 1

A 45-year-old man was diagnosed with idiopathic PAH (Table 2) without evidence of connective tissue disease or other identifiable causes of esophageal dysfunction. He was initiated on intravenous epoprostenol, sildenafil, ambrisentan, and was enrolled in the STELLAR trial testing the effect of sotatercept vs placebo on outcomes in PAH. Ultimately, he experienced further decline, withdrew from the study, and underwent a candidacy evaluation for lung transplantation.

**Table 2:** Hemodynamic Characteristics and Key Esophageal Assessment Findings in Patients With PDE5i-Associated Esophageal Dysmotility

Case	Age	Hemodynamics (mm Hg)	First manometry assessment	Second manometry assessment	Time interval
1	47	RA 5, RV 66/6, PA 65/21, mPAP 40, PAWP 6, PVR 348 dynes-sec/cm^5^	Severe ineffective esophageal motility with 80% failed and 20% weak swallows. LES pressure 13 mm Hg.	No major disorder of esophageal peristalsis with 0% failed, 40% weak, and 60% normal swallows. LES pressure 21 mm Hg.	4 months
2	61	RA 6, RV 61/11, PA 59/24, mPAP 37, PAWP 5, PVR 546 dynes-sec/cm^5^	Absent contractility with 100% failed swallows. LES pressure 12 mm Hg.	Ineffective esophageal motility with 20% failed and 60% weak swallows. LES pressure 29 mm Hg.	2 weeks

Abbreviations: LES, lower esophageal sphincter; mPAP, mean pulmonary artery pressure; PA, pulmonary artery pressure; PAWP, pulmonary artery wedge pressure; PVR, pulmonary vascular resistance; RA, right atrial pressure; RV, right ventricular pressure.

Age and hemodynamics are included from the assessment closest to the first esophageal manometry. Repeat manometry off PDE5i was notable for improvements in esophageal motility and LES pressure.

**Figure 1:**
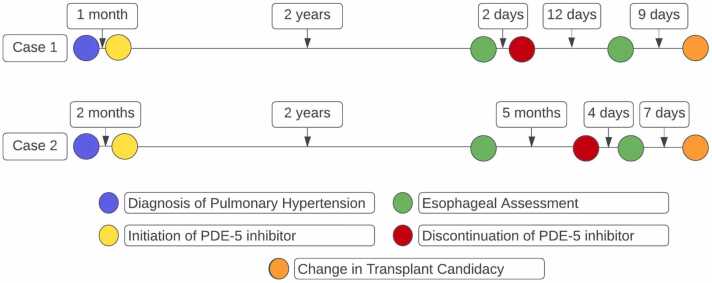
Timeline of PDE5i therapy and esophageal assessment. In 2 patients with pulmonary arterial hypertension, we identified PDE5i treatment as a reversible cause of esophageal dysmotility that had implications for lung transplant candidacy. Abbreviations: PDE5i, phosphodiesterase-type 5 inhibitor.

Esophageal manometry revealed that 80% of swallow attempts failed and 0% were normal. The study was interpreted as “severe ineffective esophageal motility,” which was a contraindication to lung transplantation at his center. Repeat manometry was performed 4 days after holding sildenafil, with ambrisentan and epoprostenol continued (Figure 1). This study showed 0% failed and 60% normal swallows. With evidence of normalized esophageal function, the study supported a diagnosis of PDE5i-induced esophageal dysmotility and excluded a primary esophageal condition that would otherwise have disqualified him for transplantation. The patient’s condition ultimately stabilized after epoprostenol dose escalation, and active transplant listing was deferred. Sildenafil was resumed and he remains eligible for listing in the future if indicated.

## Case 2

A 59-year-old man was diagnosed with idiopathic PAH (Table 2). Comorbidities included mild interstitial lung disease, multivessel coronary artery disease, and heart failure with preserved ejection fraction. He had no laboratory evidence of connective tissue disease.

He was initiated on sildenafil monotherapy shortly after diagnosis of PAH. Inhaled treprostinil was added 2 years later for worsening right ventricular failure. Due to poor tolerance of PAH therapy and progressive functional limitation, he was evaluated for lung transplantation. The patient underwent esophageal manometry as part of a pretransplant evaluation, which revealed “absent esophageal contractility” and a 100% failure rate of swallows. He was determined not to be a transplant candidate specifically due to the manometry findings. Sildenafil was held and manometry was repeated 14 days later. The second study showed only 20% failed swallows, which satisfied transplant program criteria for listing (Figure 1). Five months later, he underwent heart-lung transplantation without complications attributable to esophageal dysfunction.

## Discussion

In a single health care system, esophageal dysmotility is common in patients with PAH treated with PDE5i. We report 2 cases of PDE5i-induced esophageal dysmotility diagnosed by high-resolution manometry that were reversible after discontinuing PDE5i. Recognition of this off-target effect of PAH-specific therapy mitigated concern for a primary esophageal motility disorder, thereby re-establishing the option of lung transplantation for 2 otherwise acceptable candidates. These cases add to the experience first reported by Opotowsky et al and emphasize an overlooked iatrogenic comorbidity, which may preclude lung transplantation at centers that routinely screen patients by esophageal manometry.^7^ Collectively, these cases raise questions regarding how best to assess esophageal function in patients with PAH being evaluated for lung transplantation. The prevalence and burden of PDE5i-induced esophageal dysmotility is unknown, owing perhaps to limitations surrounding clinician awareness, the low incidence of PAH, and a lack of systematic esophageal assessment by manometry in the PAH population.

PDE5i has been shown to significantly decrease LES pressure, inhibit LES relaxation, and diminish the amplitude and velocity of peristaltic contractions throughout the esophagus in healthy volunteers and patients with achalasia.^4^, ^5^, ^6^, ^8^, ^9^ No studies have systematically reviewed the effect of these medications on esophageal contractility in patients with PAH. Furthermore, it is unknown if these effects extend to other nitric oxide pathway modulators, such as riociguat.

Contraindications for lung transplantation vary by center, and some programs report favorable outcomes in populations enriched for esophageal dysmotility, such as scleroderma.^10^ However, at centers that screen potential lung recipients by esophageal manometry, unrecognized PDE5i-associated esophageal dysfunction may jeopardize access to lung transplantation in otherwise appropriate candidates.

Although this report is limited by a small sample size and retrospective analysis, these data reinforce a possible association between PDE5i therapy and esophageal dysmotility, which has important implications for lung transplantation candidacy. Clinicians should consider the safety of repeating manometry off PDE5i therapy if esophageal dysmotility is identified as a barrier to transplant listing. As PDE5i therapy is discontinued after lung transplantation for PAH, PDE5i-associated esophageal dysmotility would not be expected to threaten lung allograft function. Prospective data collection is needed to systematically define the extent of this phenomenon, minimize iatrogenic complications of PAH therapies, and inform strategies to maximize transplant eligibility in patients with PAH on PDE5i.

## Authorship Contributions

Conceptualization, Data curation: M.S.M., S.W.J., A.R.O., M.J.L., N.S.S., H.J.G., B.A.M., B.M.W. Data analysis, Project administration, Supervision: M.S.M., S.W.J., B.A.M., B.M.W. Manuscript writing and revision: M.S.M., S.W.J., A.R.O., R.H.G., A.K.W., A.W., J.R.-L., B.A.M., B.M.W. Resources: H.J.G., N.S.S., A.K.W., A.W., J.R.-L.

## Disclosure statement

The authors declare the following financial interests/personal relationships which may be considered as potential competing interests: Bradley M. Wertheim reports a relationship with Change Healthcare Inc that includes: consulting or advisory. Bradley A. Maron reports a relationship with Actelion Pharmaceuticals US Inc. that includes consulting or advisory. Bradley A. Maron reports a relationship with Deerfield Company that includes funding grants. Bradley A. Maron reports a relationship with Tenax Therapeutics that includes consulting or advisory. Bradley M. Wertheim has patent #63/541,939 pending to Mass General Brigham. Bradley A. Maron has patent #63/541,939 pending to Mass General Brigham. Alexandra K. Wong and Alison S. Witkin report relationships with Janssen that includes consulting or advisory. There other authors declare that they have no known competing financial interests or personal relationships that could have appeared to influence the work reported in this paper.

Funding: 10.13039/100000050NHLBI 1K08HL151976 to B.M.W.
